# Supplementary material for: Predicting coarse-grained representations of biogeochemical cycles from metabarcoding data
Source: Bioinformatics. 2025 Jul 15;41(Suppl 1):i49–57. doi: 10.1093/bioinformatics/btaf230 (PMC12261419; doi:10.1093/bioinformatics/btaf230)
Supplement: btaf230_Supplementary_Data [file btaf230_supplementary_data.pdf]

Supplementary information for ”Predicting coarse-grained representations of biogeochemical cycles from metabarcoding data”

Arnaud Belcour [1,2*], Loris Megy [3], Sylvain Stephant [4], Caroline Michel [4], Sétareh Rad [4], Petra Bombach [5], Nicole Dopffel [6], Hidde de Jong [1,2] and Delphine Ropers [1,2*]

1. Univ. Grenoble Alpes, Inria, 38000 Grenoble, France
2. Université Grenoble Alpes, CNRS, LIPhy, Grenoble, France
3. Gricad, Inria, CNRS, Université Grenoble Alpes, Grenoble INP, 38000 Grenoble, France
4. French Geological Survey (BRGM), Orléans, France
5. Isodetect GmbH, Germany
6. NORCE Norwegian Research Center AS, Norway

∗ To whom correspondence should be addressed. Inria - Université Grenoble Alpes, 655 avenue de l'Europe, Montbonnot, 38334 Saint Ismier CEDEX, France. arnaud.belcour@inria.fr and delphine.ropers@inria.fr

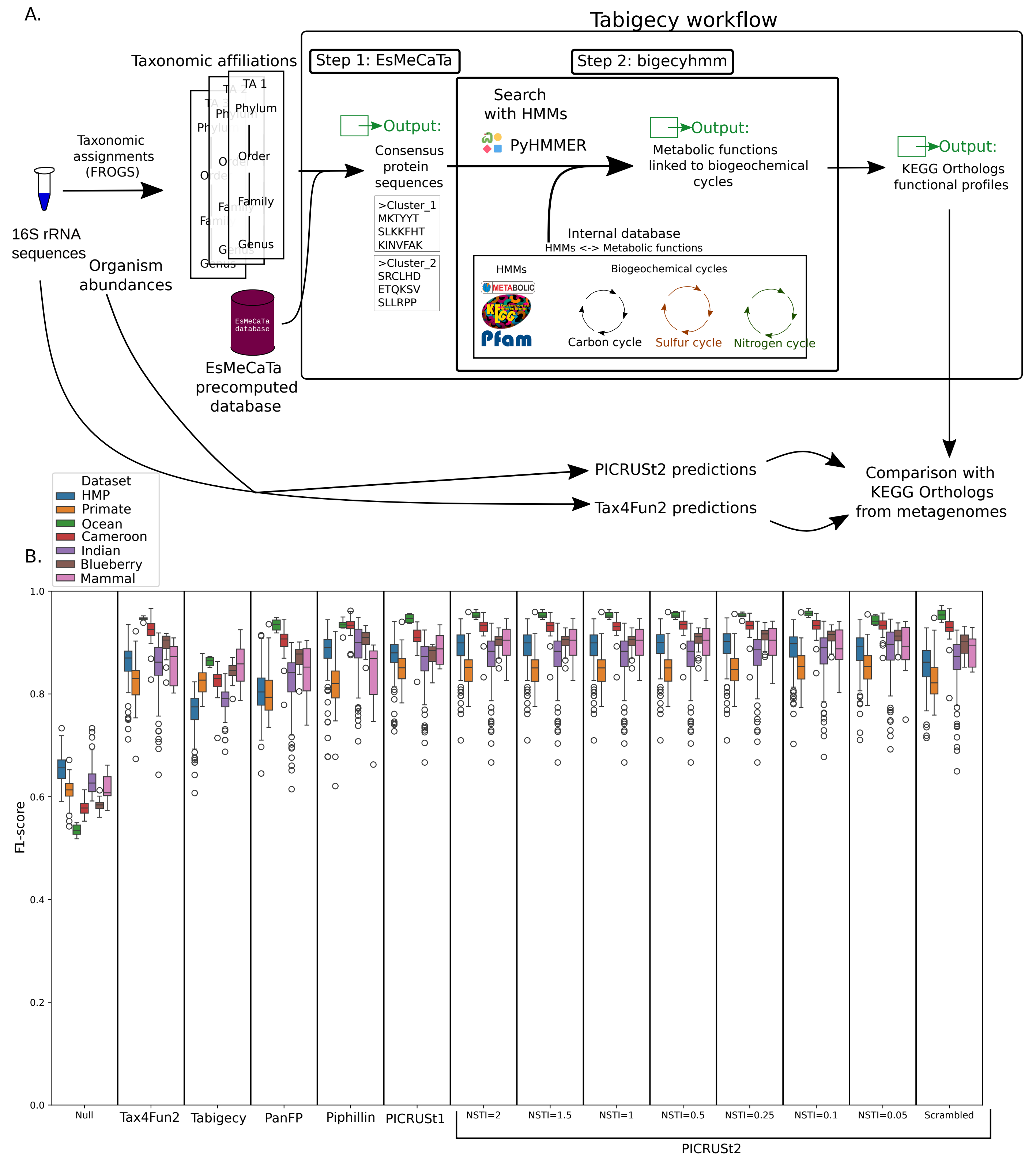

Figure S1: Comparison of predicted metabolic functions by Tabigecy *vs* PICRUSt2 and Tax4Fun2 [4, 9], using seven metabarcoding datasets. **A.** Schematic overview of the comparison. 16S rRNA sequences, abundance files and PICRUSt2/Tax4Fun2 predictions were retrieved from [4]. Taxonomic assignment of 16S rRNA sequences was performed with the FROGs pipeline [5, 1]. The resulting taxonomic affiliations were processed by means of Tabigecy. **B.** Comparison of Kegg Ortholog (KO) predictions from the three tools (PICRUSt2, Tax4Fun2 and Tabigecy) against the reference metagenomic functional profiles. The three tools are seen to give similar results on the datasets (F1 around 0.8). The scores for Tabigecy are minimal values obtained with default parameters that were not finetuned for the benchmarking datasets.

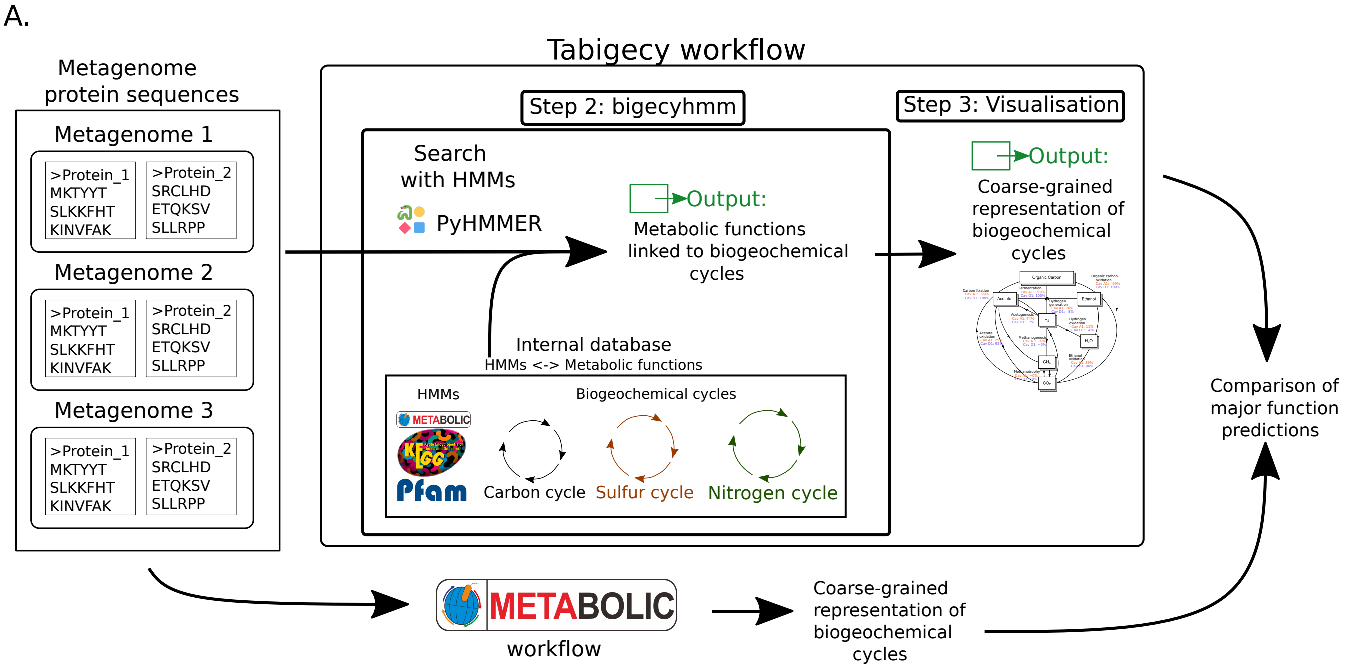

B.

Dataset from Glass et al. 2021
(30 genomes)

Biogeochemical cycle step predictions	Predicted by bigecyhmm	Not predicted by bigecyhmm
Predicted by METABOLIC	180	0
Not predicted by METABOLIC	1	715

F1-score = 0.99

Dataset from Diamond et al. 2019
(897 genomes)

Biogeochemical cycle step predictions	Predicted by bigecyhmm	Not predicted by bigecyhmm
Predicted by METABOLIC	5 193	50
Not predicted by METABOLIC	2	23 459

F1-score = 0.99

Figure S2: Comparison of predicted coarse-grained representations of biogeochemical cycles obtained by bigecyhmm *vs* METABOLIC [10], using as input two metagenomic datasets. **A.** Schematic overview of the comparison. Protein sequences from metagenomes of two datasets [3, 6] were retrieved. METABOLIC and bigecyhmm were applied to the protein sequences to predict the presence/absence of major functions in the biogeochemical cycles. **B.** Confusion matrices showing the number of functions predicted by METABOLIC and bigecyhmm. The correspondence between the results was quantified by means of the F1-score. METABOLIC and bigecyhmm are seen to give almost identical results (F1=0.99).

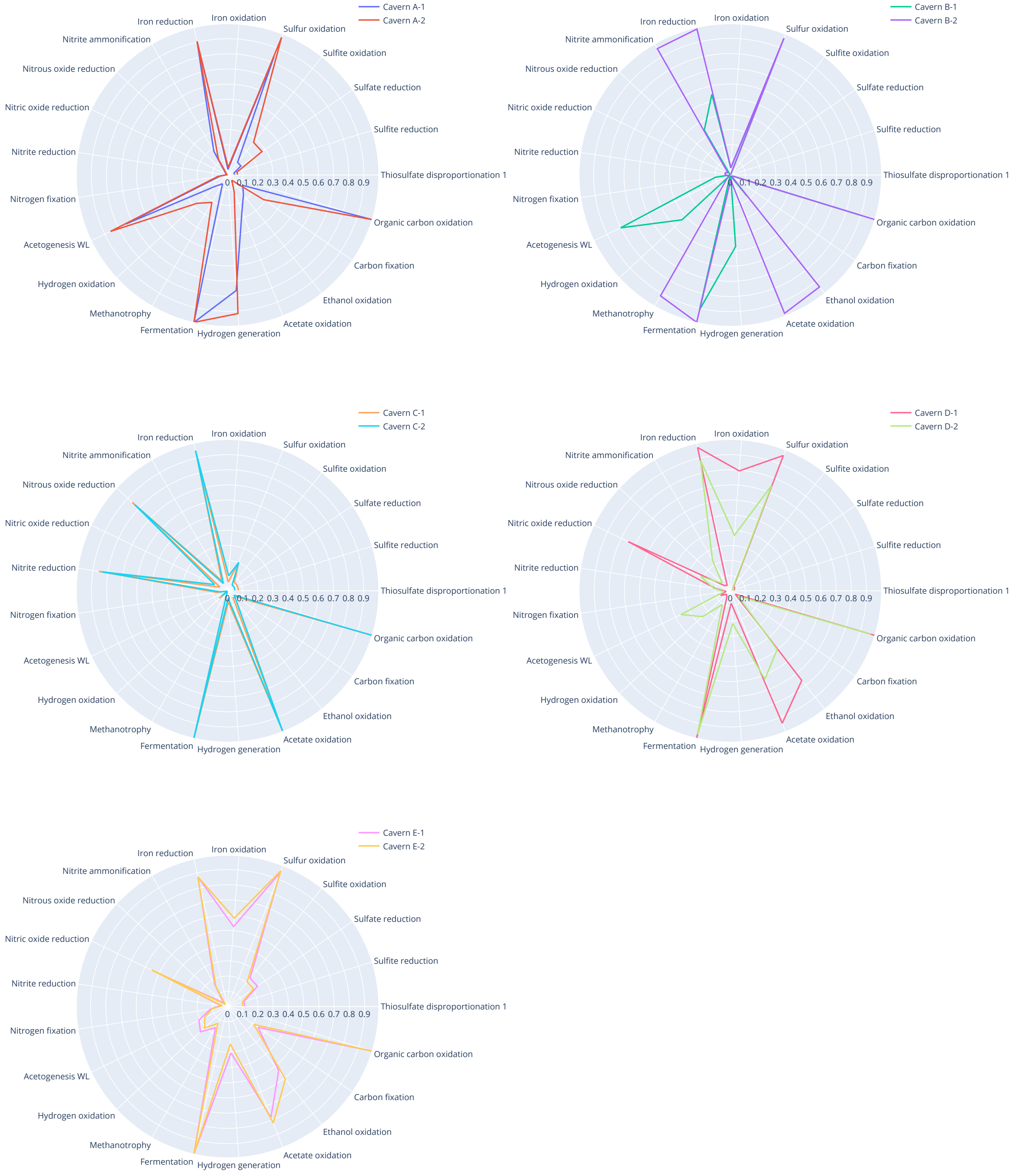

Figure S3: Polar plots showing, for each major metabolic function, the relative abundance of microorganisms found in the samples in the Schwab dataset [7]. Only functions having at least 10% of relative abundance in at least one of the samples are shown.

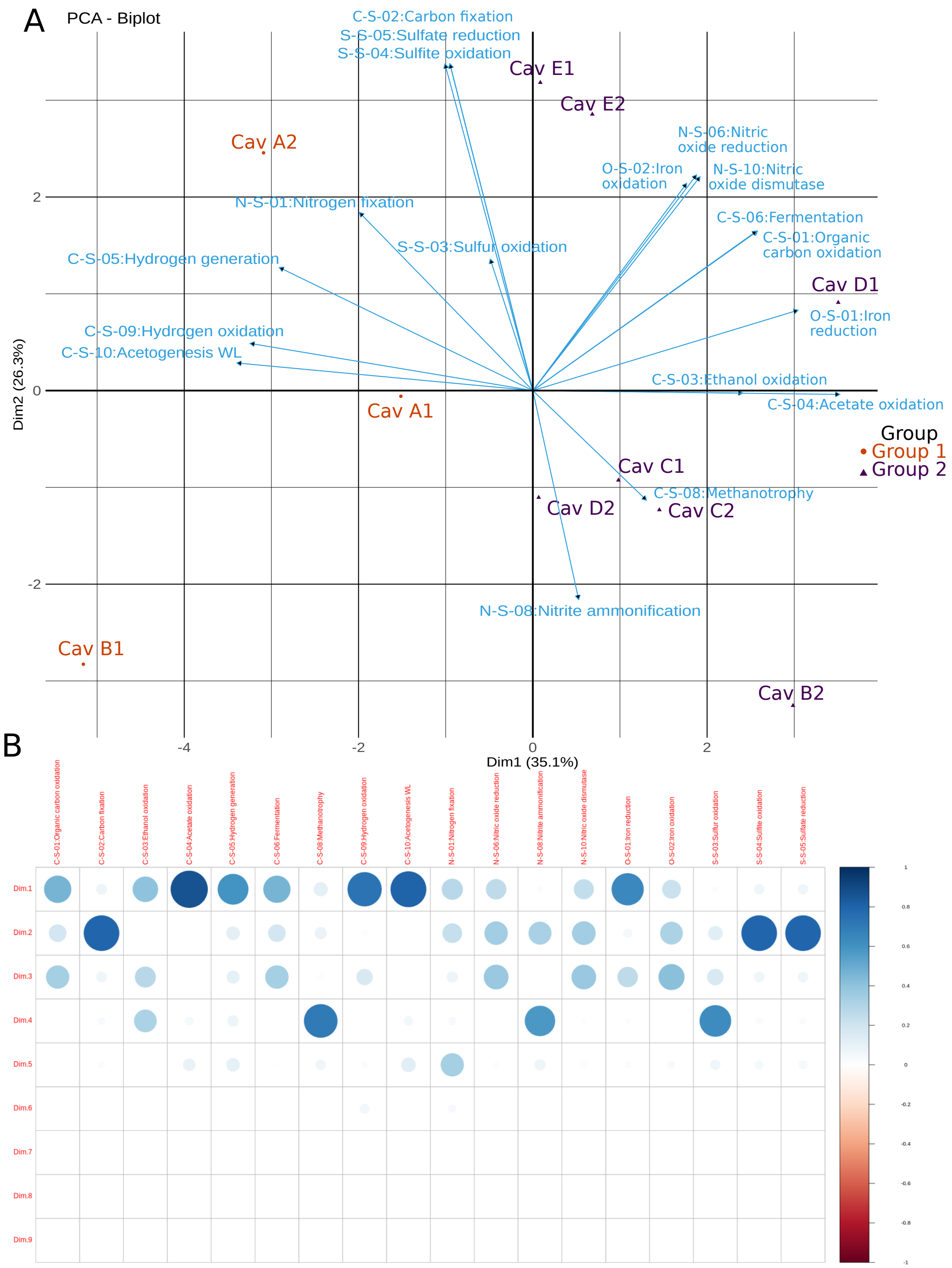

Figure S4: Principal Component Analysis (PCA) of the results obtained for the Schwab dataset. **A.** Projection on the first two PCA dimensions of the relative abundances of metabolic functions reconstructed from the samples in the Schwab dataset [7]. The vectors in the biplot represent the metabolic functions and the points the salt caverns. The latter are clustered in two groups located on opposite sides of the origin along the first PCA dimension (accounting for 35% of variance). The first group consists of samples A1, A2, and B1, the second group of the samples for the remaining caverns. Negative correlations are observed between metabolic functions. For example, the vectors representing acetogenesis and acetate oxidation point in opposite directions. **B.** Plot of the correlation of the relative abundances of metabolic functions in the Schwab dataset samples with the 9 PCA dimensions.

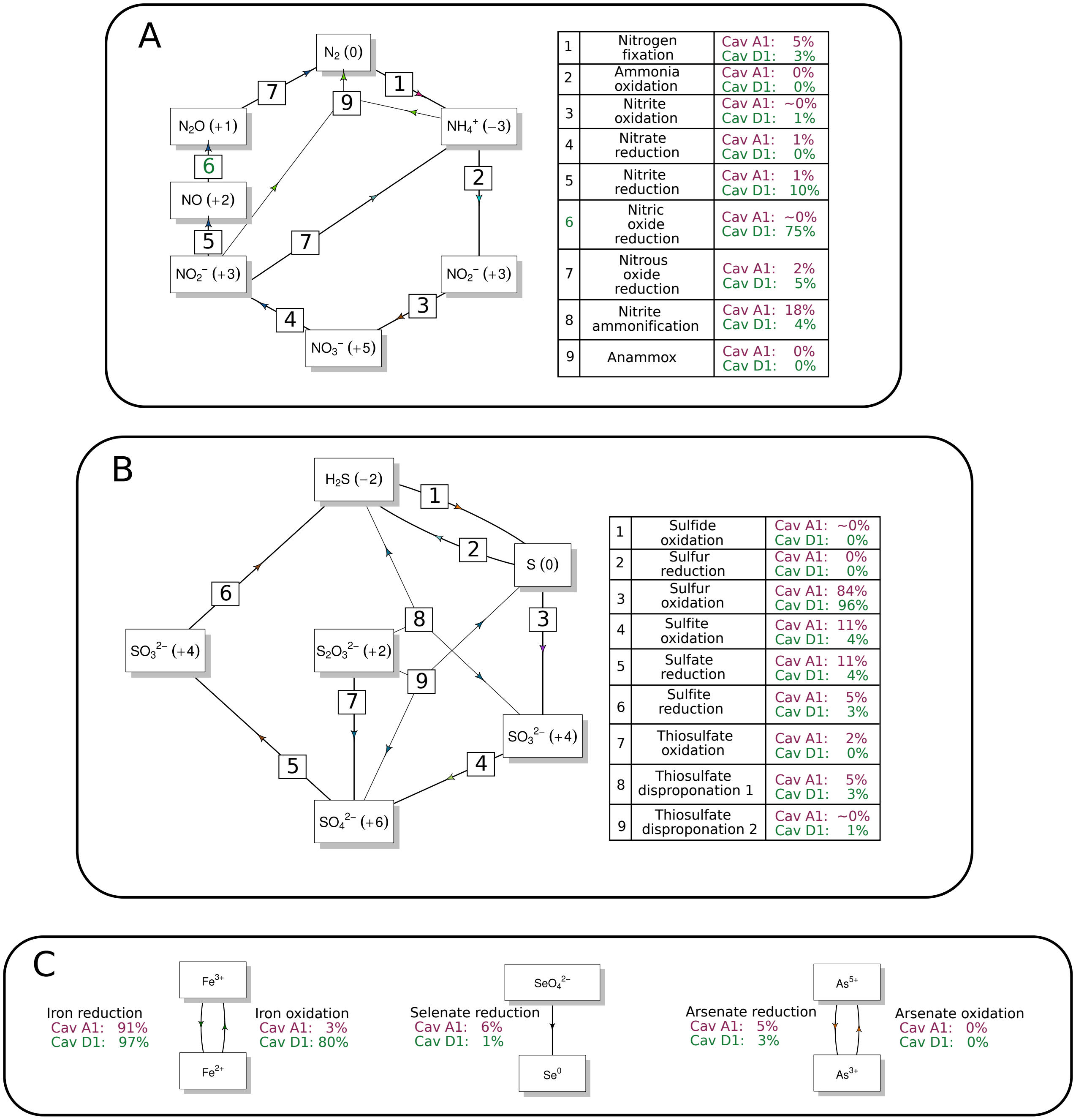

Figure S5: Projection of the metabolic functions derived for the Schwab dataset on the nitrogen (A), sulfur (B), and other cycles (C). Like the carbon cycle diagram in Fig. 2B in the main text, the diagrams are taken from [10]. The diagrams are completed with weights of the functions, given by the relative abundances of the microorganisms in the two considered samples of the Schwab dataset (Cav. A1 and Cav. D1).

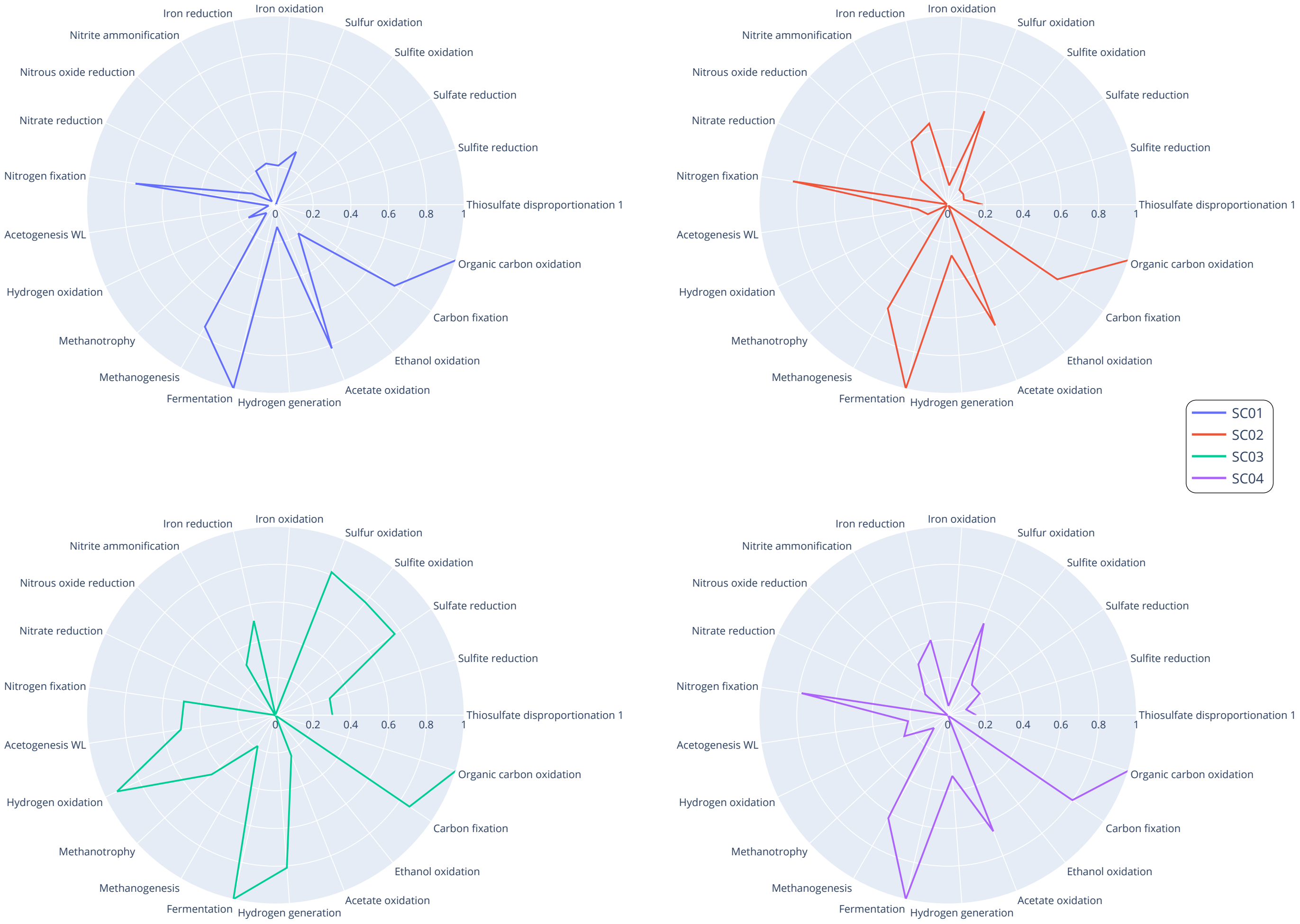

Figure S6: Polar plots showing, for each major metabolic function, the relative abundance of microorganisms found in the samples in the Bordenave dataset [2]. Only functions having at least 10% of relative abundance in at least one of the samples are shown.

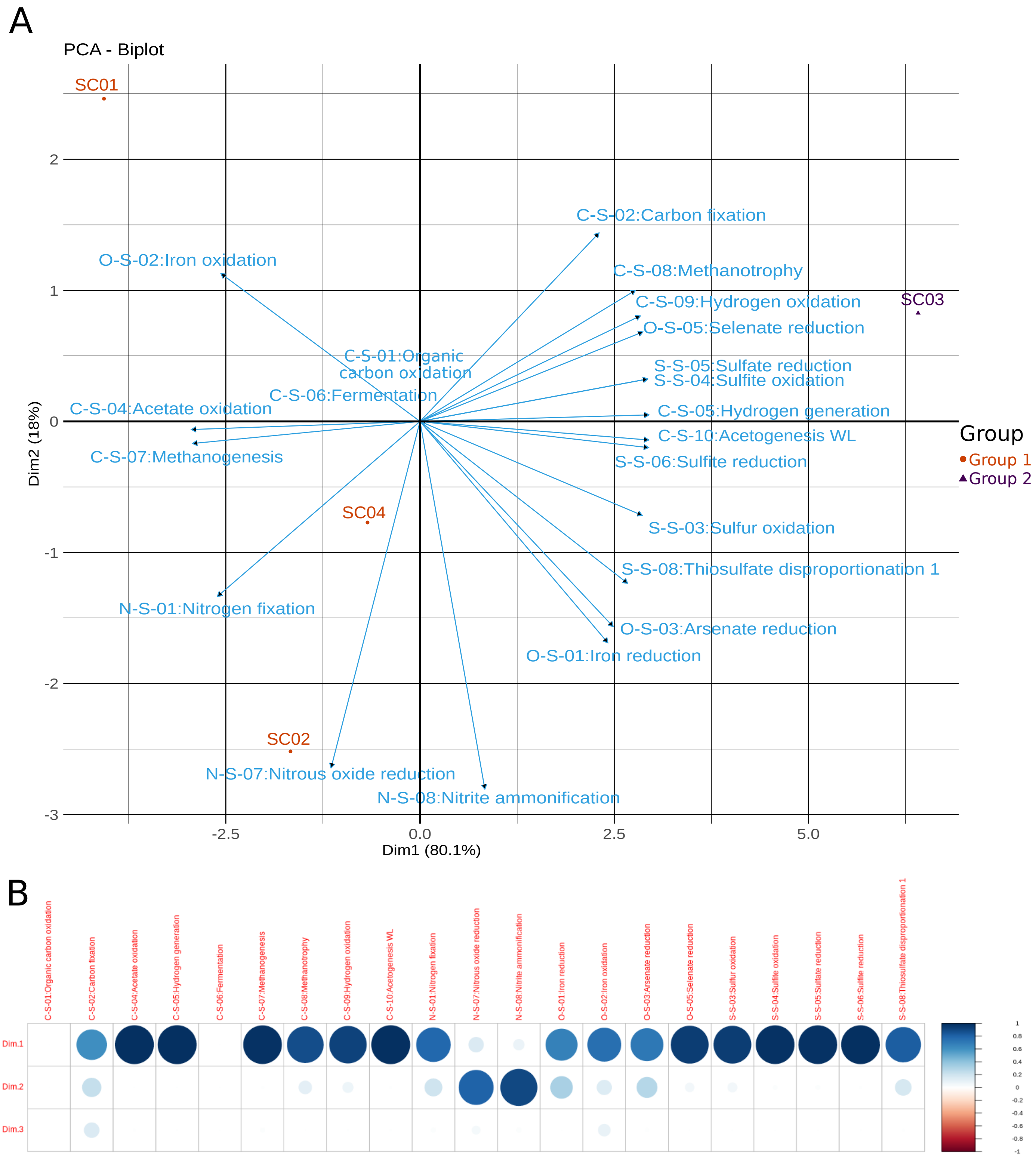

Figure S7: Principal Component Analysis (PCA) of the results obtained for the Bordenave dataset. **A.** Projection on the first two PCA dimensions of the relative abundances of metabolic functions reconstructed from the samples in the Bordenave dataset [2]. The vectors in the biplot represent the metabolic functions and the points the salt caverns. The latter are clustered in two groups located on opposite sides of the origin along the first PCA dimension (accounting for 80% of the variance). The first group consists of samples SC01, SC02, and SC04, the second group of the sample for the remaining cavern SC03. Negative correlations are observed between metabolic functions. For example, the vectors representing acetogenesis and acetate oxidation point in opposite directions. **B.** Plot of the correlation of the relative abundances of metabolic functions in the Bordenave dataset samples with the 3 PCA dimensions.

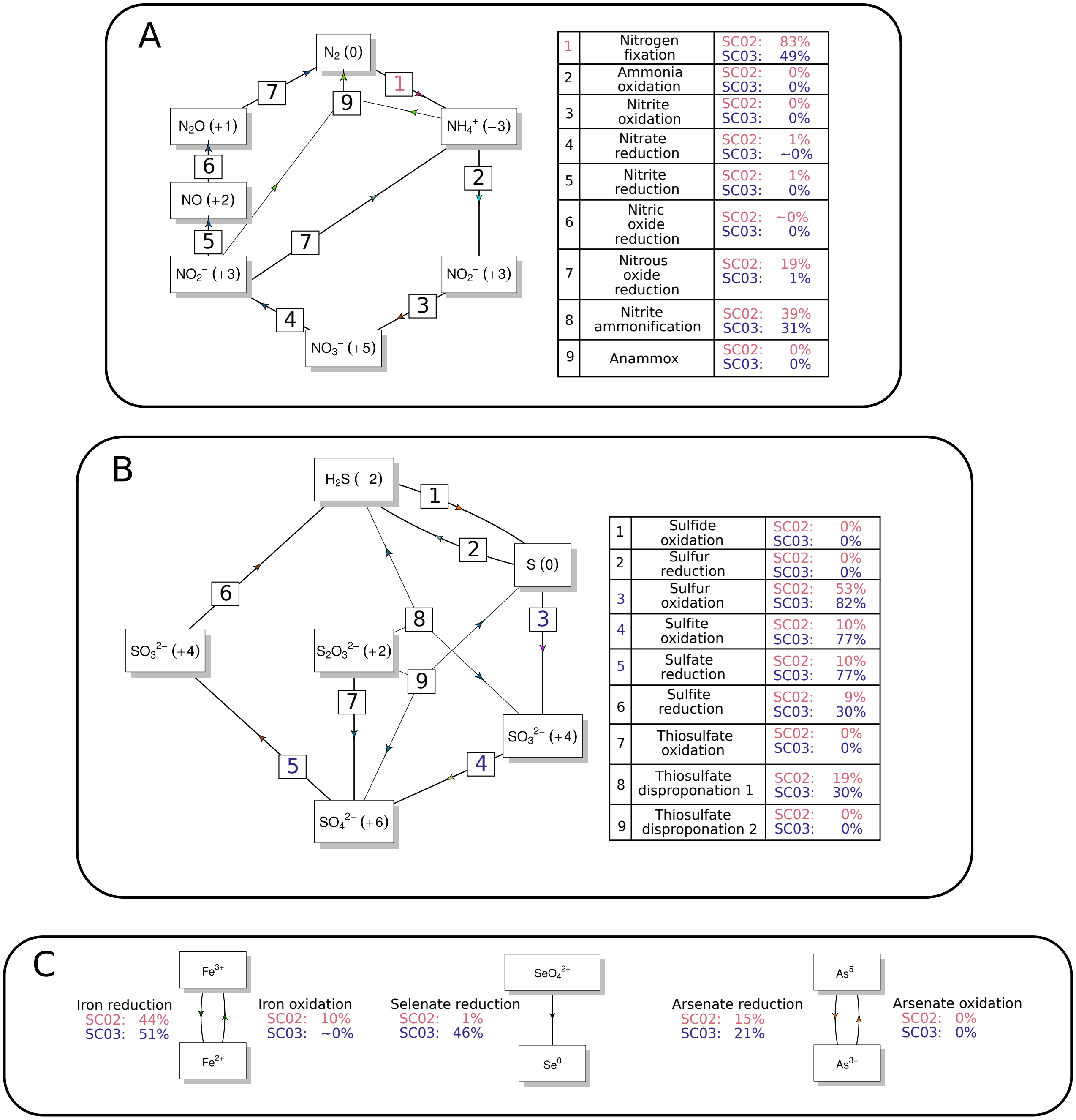

Figure S8: Projection of the metabolic functions derived for the Bordenave dataset on the nitrogen (A), sulfur (B), and other cycles (C). Like the carbon cycle diagram in Fig. 2D in the main text, the diagrams are taken from [10]. The diagrams are completed with weights of the functions, given by the relative abundances of the microorganisms in the two considered samples of the Bordenave dataset (SC02 and SC03).

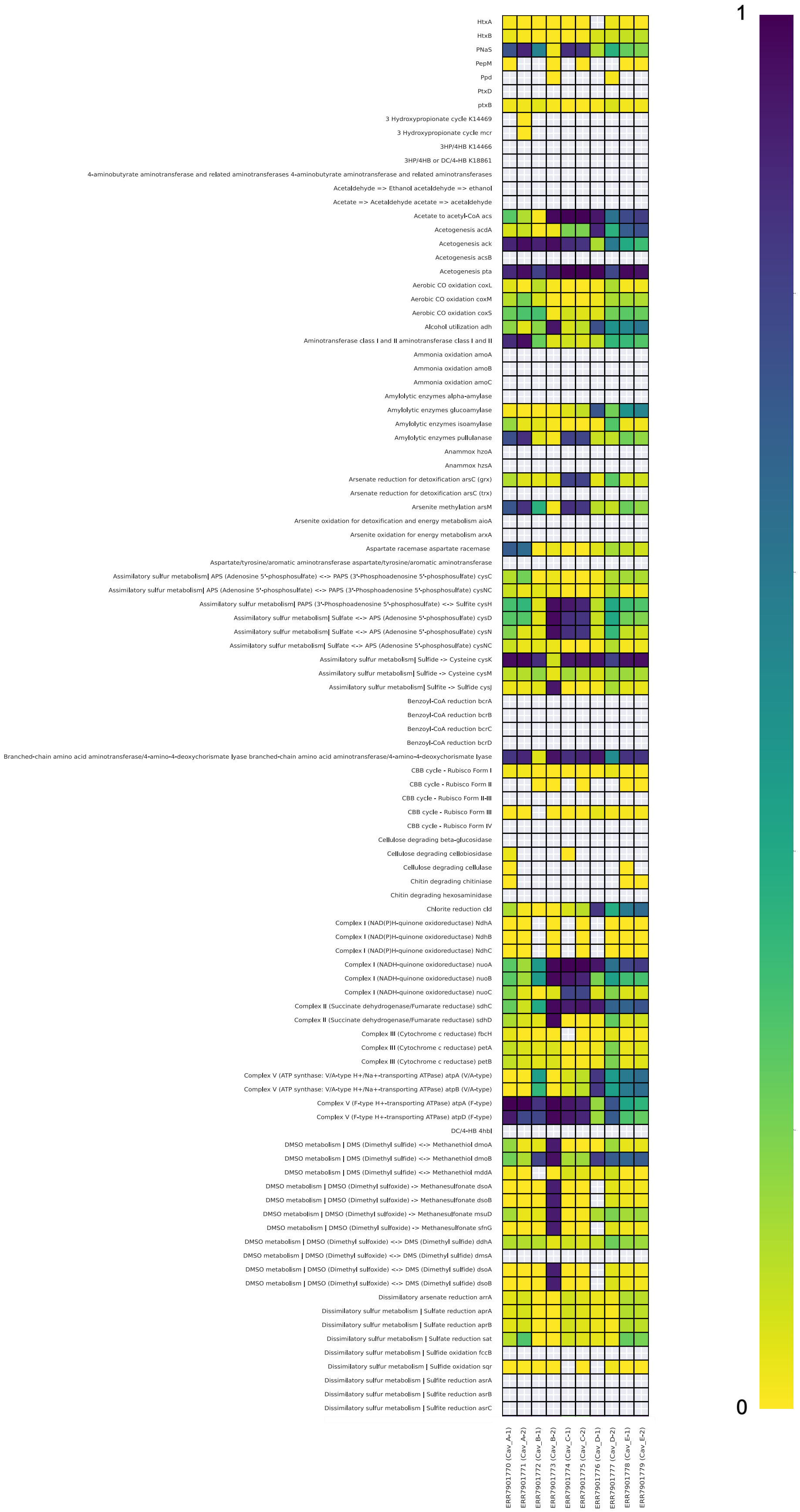

Figure S9: Heatmap showing, for each metabolic function, the relative abundance of microorganisms found in the samples in the Schwab dataset.

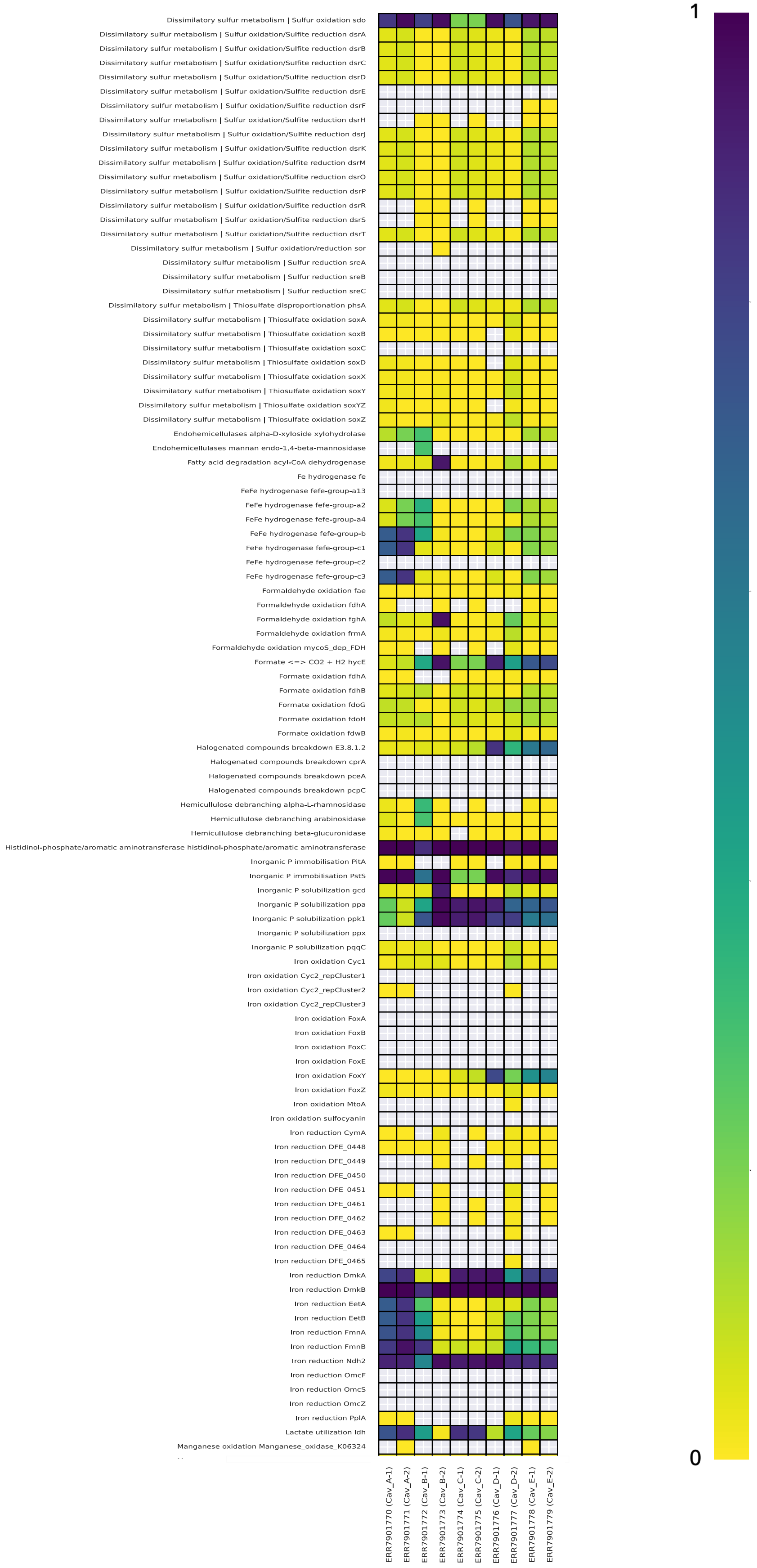

Figure S9: (Continued)

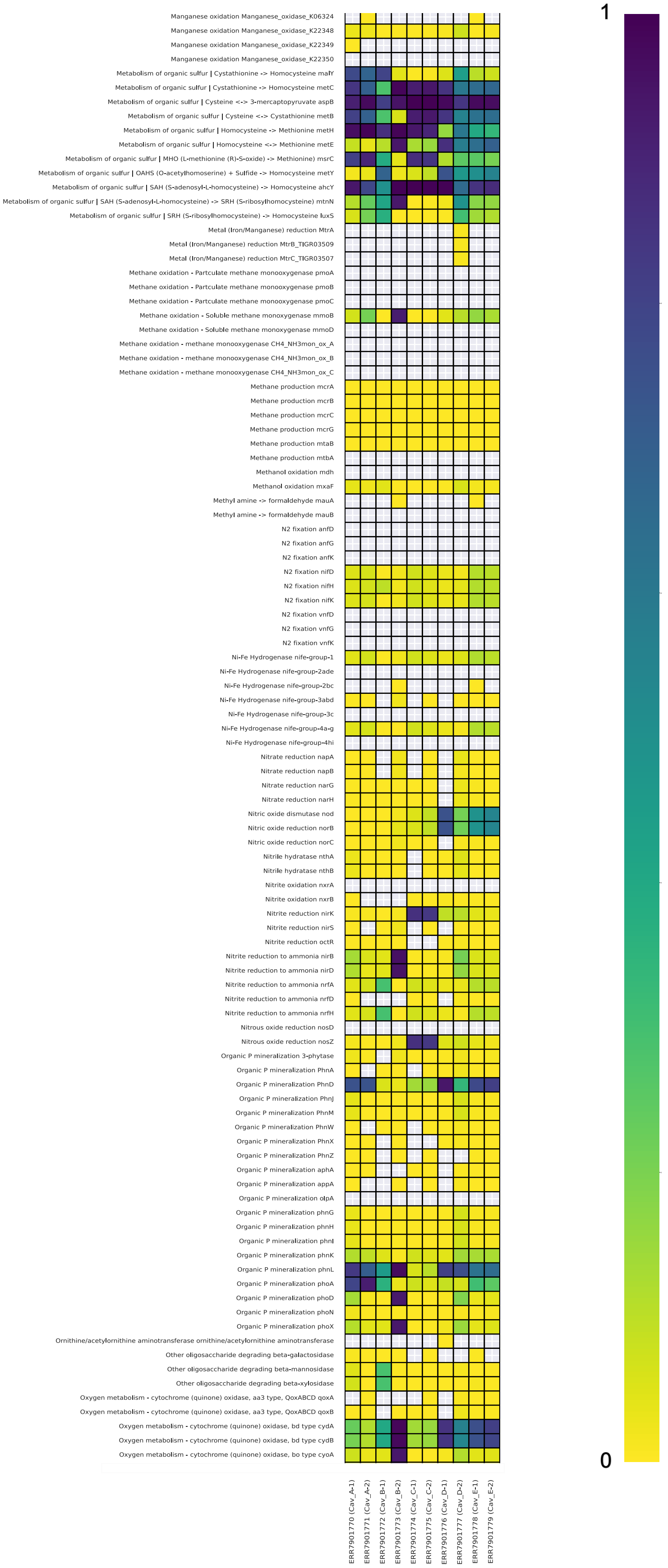

Figure S9: (Continued)

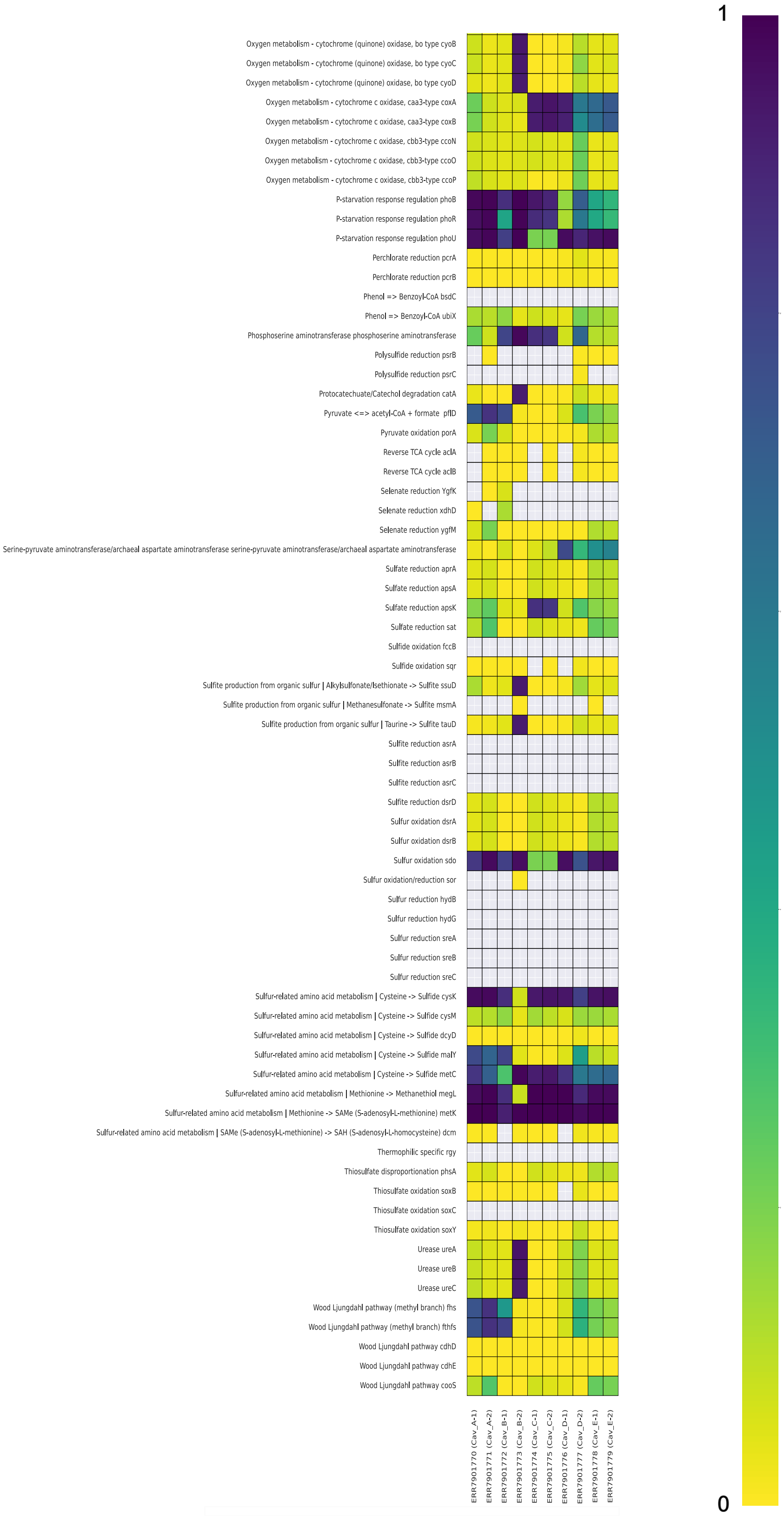

Figure S9: (Continued)

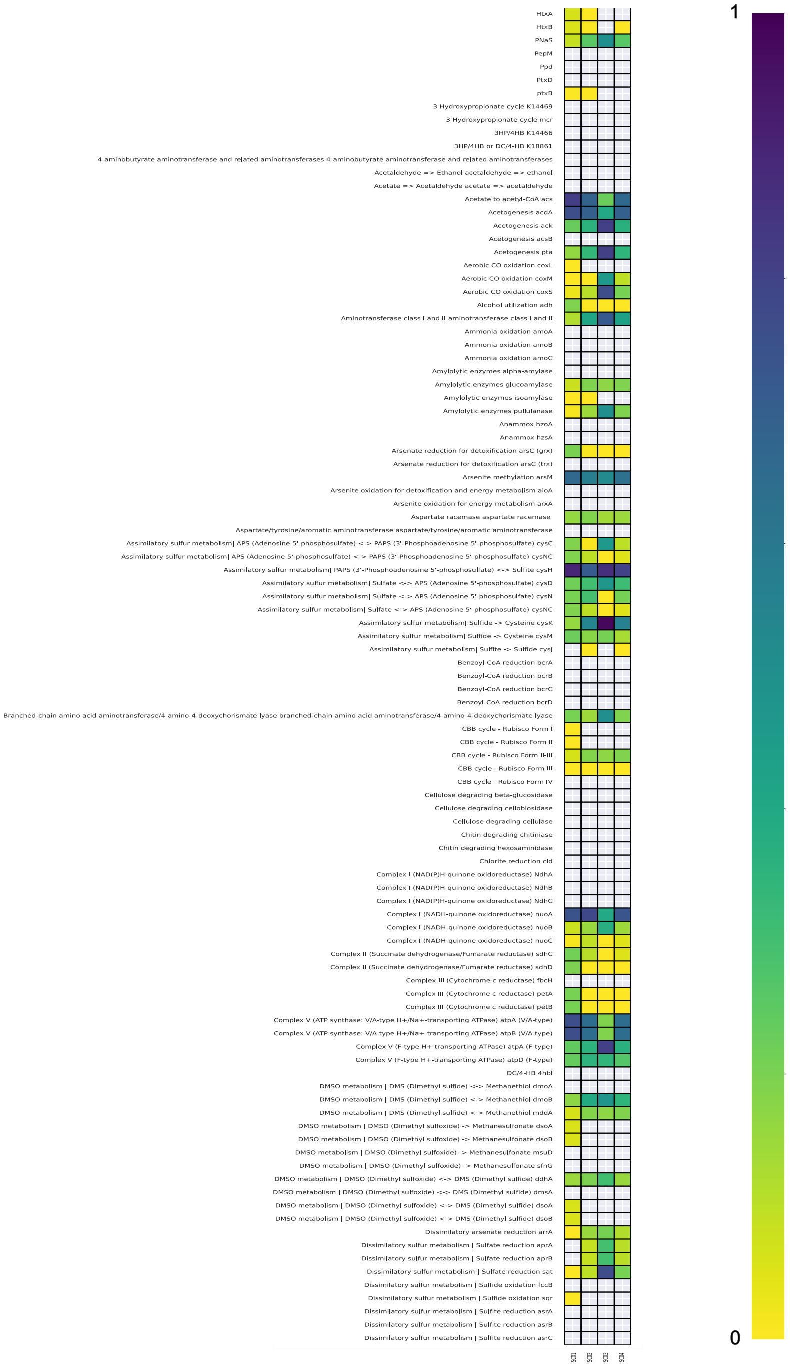

Figure S10: Heatmap showing, for each metabolic function, the relative abundance of microorganisms found in the samples in the Bordenave dataset.

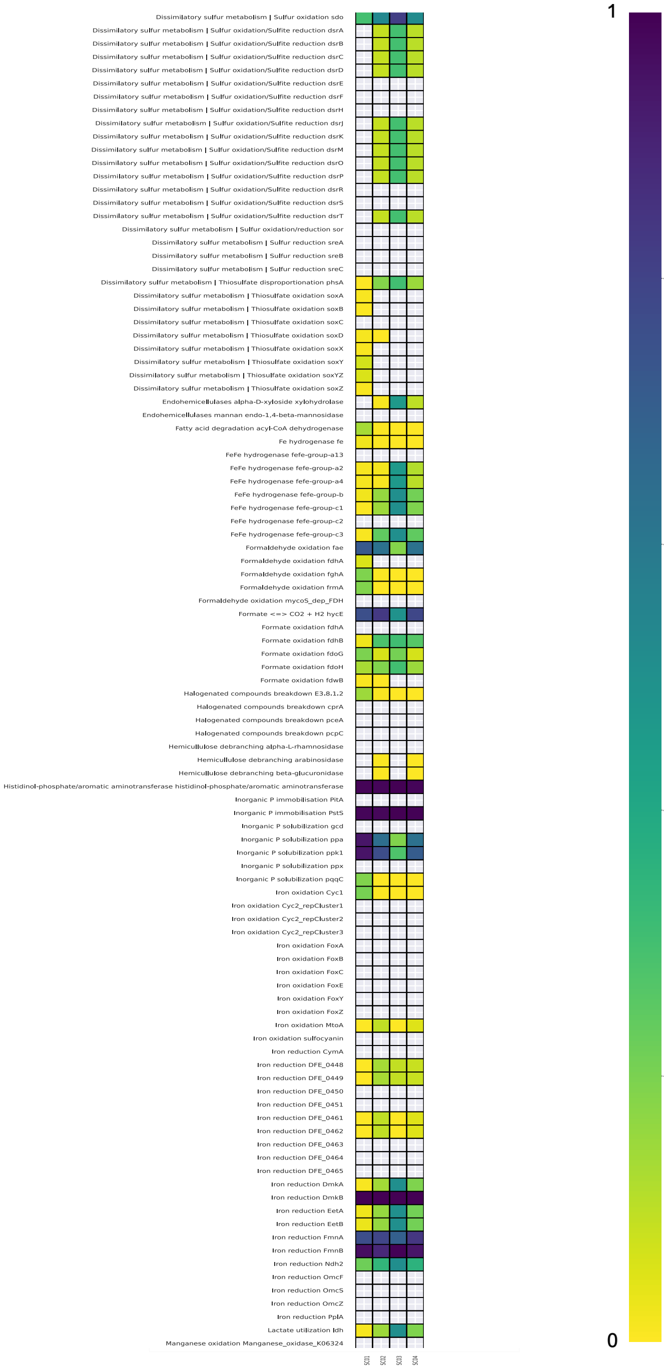

Figure S10: (Continued)

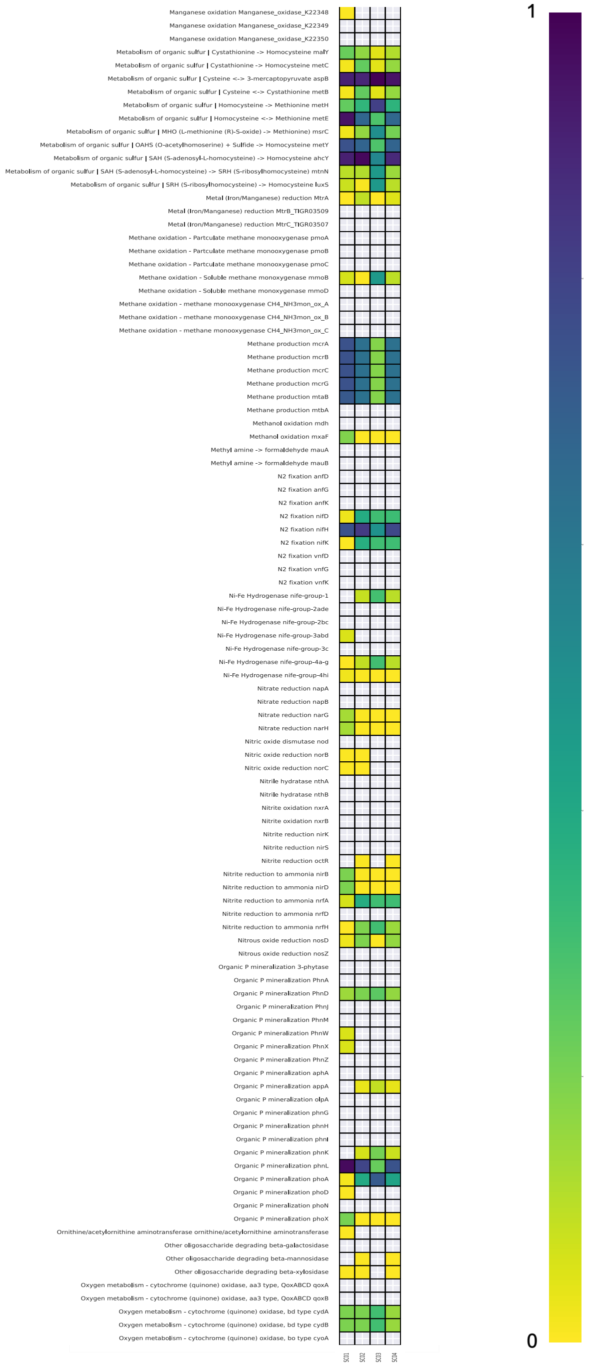

Figure S10: (Continued)

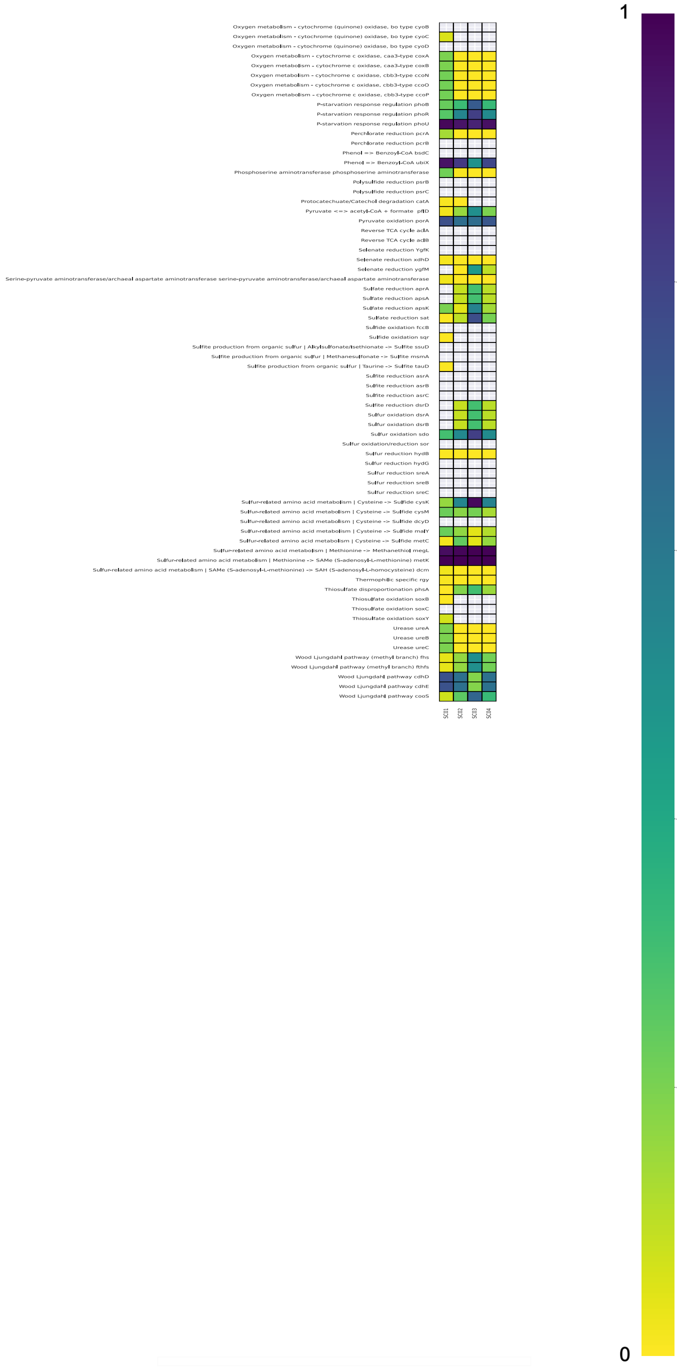

Figure S10: (Continued)

Supplementary Text S1: Benchmarking of Tabigecy subtasks

Comparison with PICRUST2 and Tax4Fun2

We compared the prediction of metabolic functions by means of Tabigecy with the results of PICRUSt2 and Tax4Fun2 [4, 9], using seven metabarcoding datasets from the PICRUSt2 article [4]. These datasets include 16S rRNA sequences and their associated abundances for different microbiota (ocean, human, mammal, primate). The input data were retrieved from `https://github.com/gavinmdouglas/picrust2_manuscript`. The taxonomic assignment of the 16S rRNA sequences was processed by means of the FROGs pipeline (version 4.1.0; [5, 1]) accessible through Galaxy France (`https://usegalaxy.fr/`; [8]). Tabigecy version 0.1.1 (with EsMeCaTa 0.6.4 and bigecyhmm 0.1.6) with default parameters was applied to the inferred taxonomic affiliations using the abundance files associated with the 16S rRNA sequences. The Kegg Orthologs (KOs) predicted by the three tools (PICRUSt2, Tax4Fun2 and Tabigecy) were compared with the KOs from the reference metagenomic profiles. More precisely, for each of the datasets, we quantified the presence/absence of predicted KOs with respect to their presence/absence in the metagenomic profiles using the F1-score. The results are shown in Supplementary fig. S1.

Comparison with METABOLIC

We compared the predicted coarse-grained representations of biogeochemical cycles obtained by bigecyhmm with the predictions of METABOLIC [10] using as input two metagenomic datasets [3, 6]. The protein sequences of the metagenomes were retrieved and processed by means of METABOLIC and bigecyhmm version 0.1.6 (10 cores). The predicted functions (inputs for the nutrient cycling diagrams generated by METABOLIC) were retrieved and compared in confusion matrices for the two datasets. An F1-score was computed from these matrices to quantify the correspondence between the predictions of Tabigecy and METABOLIC (Supplementary fig. S2).

References

[1] M. Bernard, O. Rué, M. Mariadassou, et al. FROGS: a powerful tool to analyse the diversity of fungi with special management of internal transcribed spacers. *Brief Bioinform*, 22(6):bbab318, 2021.

[2] S. Bordenave, I. Chatterjee, and G. Voordouw. Microbial community structure and microbial activities related to CO2 storage capacities of a salt cavern. *Int Biodeterior Biodegradation*, 81:82–7, 2013.

[3] S. Diamond, P. F. Andeer, Z. Li, A. Crits-Christoph, D. Burstein, K. Anantharaman, K. R. Lane, B. C. Thomas, C. Pan, T. R. Northen, and J. F. Banfield. Mediterranean grassland soil C–N compound turnover is dependent on rainfall and depth, and is mediated by genomically divergent microorganisms. *Nat. Microbiol.*, 4(8):1356–1367, 2019.

[4] G. M. Douglas, V. J. Maffei, J. R. Zaneveld, et al. PICRUSt2 for prediction of metagenome functions. *Nat Biotechnol*, 38(6):685–8, 2020.

[5] F. Escudié, L. Auer, M. Bernard, et al. FROGS: Find, rapidly, OTUs with Galaxy solution. *Bioinformatics*, 34(8): 1287–94, 2018.

[6] J. B. Glass, P. Ranjan, C. B. Kretz, B. L. Nunn, A. M. Johnson, M. Xu, J. McManus, and F. J. Stewart. Microbial metabolism and adaptations in Atribacteria-dominated methane hydrate sediments. *Environ. Microbiol.*, 23(8):4646–4660, 2021.

[7] L. Schwab, D. Popp, G. Nowack, et al. Structural analysis of microbiomes from salt caverns used for underground gas storage. *Int J Hydrogen Energy*, 47(47):20684–94, 2022.

[8] The Galaxy Community. The Galaxy platform for accessible, reproducible, and collaborative data analyses: 2024 update. *Nucleic Acids Res*, 52(W1):W83–94, 2024.

[9] F. Wemheuer, J. A. Taylor, R. Daniel, et al. Tax4Fun2: prediction of habitat-specific functional profiles and functional redundancy based on 16S rRNA gene sequences. *Environ Microbiome*, 15(1):11, 2020.

[10] Z. Zhou, P. Q. Tran, A. M. Breister, et al. METABOLIC: high-throughput profiling of microbial genomes for functional traits, metabolism, biogeochemistry, and community-scale functional networks. *Microbiome*, 10(1):33, 2022.
